# Energy and time optimal trajectories in exploratory jumps of the spider *Phidippus regius*

**DOI:** 10.1038/s41598-018-25227-9

**Published:** 2018-05-08

**Authors:** Mostafa R. A. Nabawy, Girupakaran Sivalingam, Russell J. Garwood, William J. Crowther, William I. Sellers

**Affiliations:** 1 School of Mechanical, Aerospace and Civil Engineering, The University of Manchester, Manchester, M13 9PL UK; 2 School of Earth and Environmental Sciences, The University of Manchester, Manchester, M13 9PL UK; 3 Department of Earth Sciences, The Natural History Museum, Cromwell Road, London, SW7 5BD UK

## Abstract

Jumping spiders are proficient jumpers that use jumps in a variety of behavioural contexts. We use high speed, high resolution video to measure the kinematics of a single regal jumping spider for a total of 15 different tasks based on a horizontal gap of 2–5 body lengths and vertical gap of +/−2 body lengths. For short range jumps, we show that low angled trajectories are used that minimise flight time. For longer jumps, take-off angles are steeper and closer to the optimum for minimum energy cost of transport. Comparison of jump performance against other arthropods shows that *Phidippus regius* is firmly in the group of animals that use dynamic muscle contraction for actuation as opposed to a stored energy catapult system. We find that the jump power requirements can be met from the estimated mass of leg muscle; hydraulic augmentation may be present but appears not to be energetically essential.

## Introduction

Jumping is a unique form of animal locomotion in which a rapid extension of the legs in contact with the ground provides sufficient impulse for significant airborne translation. In animal locomotion studies, the default assumption is that animals evolve for energetic efficiency^1^ but with the realisation that this assumption is often violated because clearly some animals have evolved locomotor specialisation for other functions such as high speed^2^ or prey capture^3^. Jumping was one of the first forms of locomotion to be used as a model for scaling^4^, and its theoretical optima can be calculated for jumping different distances and heights^5^. The mechanics of jumping have been extensively studied in a wide range of animals where it is used for a number of different functions: lateral progression^6^, escape^7^, prey capture^8^, and flight initiation^9^.

The jumping spiders (Araneae: Salticidae) are instantly recognisable arachnids. The group possesses sizeable anterior median eyes that dominate their square prosoma, relatively short legs (often with conspicuous markings), and they use both tracheae and book lungs for breathing^10^. The family comprises over 5,900 species, making it one of the largest spider families^11^, and is so called because these small (3–10 mm) spiders hunt by jumping. They turn to face their prey, and then effect prey capture using jumps of up to 160 mm^10^. They also jump to escape from threats^10^. Members of the family are found worldwide in many different habitats such as grassland and forest^12^. Despite the salticids’ diversity, their morphological disparity^13^, diverse predatory strategies^14^, complex behaviour^15^, and the importance of spiders to ecosystems as predators^16^, the group’s eponymous behaviour has seldom been studied in detail.

Spiders jump for different reasons. Weihmann *et al*.^17^ reported two different jump types in the Central American hunting spider *Cupiennius salei*. Unprepared jumps allowed the spider to evade a disturbance by jumping in any direction as an escape reaction. In contrast, prepared jumps were anteriorly directed, and always had a characteristic posture and motion: a backwards and downwards preliminary countermovement of the centre of mass, then a jump executed by extension of initially the fourth leg pair, then the second leg pair. Salticids are one of the most prominent and proficient jumpers, and their jumps have been documented by Ehlers^18^, and Parry and Brown^19^, and summarised by Foelix^10^. Parry and Brown^19^ assessed the jumping performance of *Sitticus pubescens* jumping across a horizontal gap of approximately 50 mm (around 10 body lengths) reporting a take-off speed of 0.67 m/s, and acceleration of 5.23 g. Hill^20^ conducted a comprehensive study assessing several aspects of the jumping performance of the spider *Phidippus princeps*. Several jumping platforms were used allowing the control of jumping horizontal distance and downwards inclination. Hill reported a maximum horizontal jump distance of 60 mm (around 4 body lengths) for which a take-off speed of 0.83 m/s, and an acceleration of 5.24 g were measured.

In a salticid jump, the front limbs are utilised in landing and prey capture, and are lifted prior to a jump^21^. The spider also uses its opisthosoma to attach a silk line to the substrate before jumping, which acts as a safety line in case of a missed landing and may act as a mechanism for directional stability in flight^22^. The limb pairs providing motive force for take-off varies between species - it can be the third, the fourth, or a combination of the two^10^. Leg extension during the acceleration phase of the jump is primarily due to straightening at the femur-patella hinge joint^19^. Foelix^10^ suggests that this straightening stems from a spike in haemolymph pressure coupled with a relaxation of flexor muscles. The work of Parry and Brown^19^ was the first to investigate any such link in jumping spiders. The authors built on prior work showing a relationship between the haemolymph pressure and extension torque at the hinge joints of the house spider *Tegenaria atrica*^23^. Parry and Brown^19^ used this relationship to estimate the acceleration of the jumping spider *S. pubescens* during take-off, calculate propulsive force and thus torques at the hinge joints, and then estimate the pressure required to cause this. They conclude that hydraulic forces are involved in the jump, in part because the estimated pressure is within a factor of two of pressures observed in the house spider, and in part due to the observation that leg spines become erect at take-off, which they propose reflects increased haemolymph pressure. In contrast, Weihmann *et al*.^17^ suggest that in hunting spiders, the jump is powered by the interaction of musculature and hydraulic mechanisms in the fourth leg pair. Some of the same authors have proposed a similar mechanism for the hind limbs of larger cursorial spiders^24^. At take-off in salticids, there is typically a positive angular velocity component about the pitch axis that causes the spider to rotate, ultimately leading to a head up attitude at landing. For jumps at a shallow angle, this attitude maximises the projected area of the limbs at landing, which presumably aids prey capture. Tension in the safety line will tend to counter body rotation in pitch and yaw. It is possible that increased tension in the safety line towards the end of the jump could produce a negative nose-down pitching moment, and reduce the attitude of the body prior to landing^22^.

The main aim of the present study is to provide deeper understanding of the mechanics of jumps, and how the jumping spider *P. regius* adapts its jumping style depending on the jumping task it is presented with. Particularly, we are interested in how choices affect the energy and time efficiency of the jump. Our hypothesis is that jumping spiders will adapt their trajectory planning depending on the nature of the jumping task. Our expectation is that they will use energetically optimised (minimum energetic cost of transport) jumps for crossing larger gaps, however that for smaller gaps more consistent with the range over which prey capture normally takes place, the trajectories will be biased towards time optimal (minimum time of flight) jumps. We consider here for the first time, both ascending and descending jumps. Whilst previous work assessed level^19,20^ and descending^20,22^ jumps for other spider species, ascending jumps have not been investigated. As an additional attribute of our work, the jumping motion is constrained to a predefined vertical plane, which reduces optical measurement uncertainty compared to previous studies^19^ where a larger freedom in the lateral landing location means the jumping plane is ill defined *a priori*. We also set the work in the broader framework of jumping in other arthropods by detailed comparison of jumping performance metrics across a large range of species, and for different jumping actuation strategies. Finally, we are able to comment on the ongoing debate of the role of hydraulic versus muscular actuation for the present spider species. As a note on terminology, the words leap and jump are used interchangeably in the literature to mean the same thing; here, we will use the word jump throughout.

## Results

### Spider morphology

A high-resolution CT scan was conducted to obtain a complete geometric description of the spider, from which we could estimate the centre of mass and confirm the leg anatomy. The morphological data provided in the Supplementary Files S1 will also allow future development of more sophisticated kinematic models that can be used for motion reconstruction and biomechanical analysis going forward.

**Figure 1:**
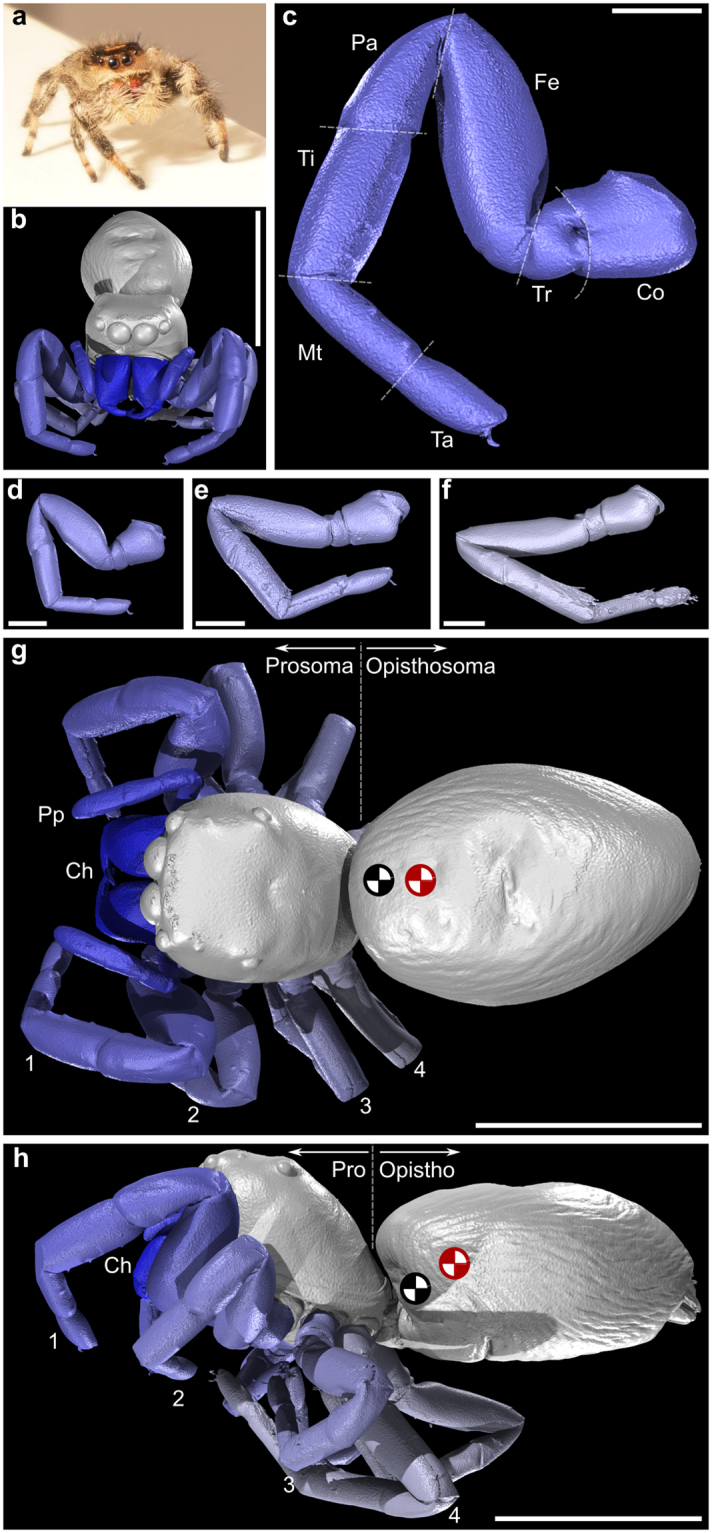
The morphology of the jumping spider *Phidippus regius*. (**a**) The individual used for the study of jumping performance. (**b**–**h**) Digital visualisations of a second specimen derived through X-ray microtomography: (**b**) Anterior view. (**c**) The first (anterior-most) walking leg. (**d**–**f**) The second, third and fourth walking legs, respectively. (**g**) Dorsal view. (**h**) Side view. Abbreviations: 1–4: walking legs 1–4; Ch: chelicerae; Co: coxa; Fe: femur; Mt: metatarsus; Pa: patella; Pp: pedipalps; Ta: tarsus; Ti: tibia; Tr: trochanter. Scale bars: **b,g,h**: 5 mm; (**c**–**f**) 1 mm. Red symbol denotes centre of mass of body alone whereas the black symbol denotes the centre of mass of the whole spider with the appendages in the position shown.

X-ray microtomography reveals the morphology of a female *P. regius* specimen in detail, Fig. 1. The body is divided into two sections (or tagma) - the anterior prosoma, and posterior opisthosoma. The former is 4.1 mm long in the scanned specimen and bears prominent eyes and the appendages including the chelicerae, pedipalps and the legs (Fig. 1b,g and h). The latter (length 7.6 mm) is separated from the prosoma by a narrow, flexible portion of the body (the pedicel), and has the openings to the respiratory organs, and the silk-producing spinnerets towards the posterior. The morphology of the legs is of the greatest relevance to the present study (Fig. 1c–f). The legs comprise seven segments. From proximal to distal these are: the coxa, trochanter, femur, patella, tibia, metatarsus and tarsus (Fig. 1c). The length of these segments, measured from the scanned specimen, are provided in Table 1; leg 1 (10.4 mm total length) and 4 (10.5 mm) are the longest, with leg 2 and 3 both totalling 8.4 mm in length. The legs terminate with a claw. For completeness, a VAXML model^25^ of the spider (stl meshes tied together with an XML file), the original volume and a Drishti Prayog volume^26^, are provided as Supplementary Files S1.

**Table 1:** Measurements of the leg segment length derived from the microtomography scan of a female *Phidippus regius* specimen.

Leg	Total length (mm)	Coxa (%)	Trochanter (%)	Femur (%)	Patella (%)	Tibia (%)	Metatarsus (%)	Tarsus (%)
1	10.4	10.6	5.8	25.9	17.3	18.3	12.5	9.6
2	8.4	10.7	5.9	28.6	15.5	16.7	11.9	10.7
3	8.4	9.5	7.1	28.6	13.1	14.3	15.5	11.9
4	10.5	7.6	6.7	27.6	9.5	20	18.1	10.5

Length of each segment is provided as a ratio of the total leg length.

The centre of mass of the spider was calculated from the CT scan volume data assuming constant density. In practice, the location of the centre of mass will vary with appendage positioning, however the appendages (including legs, chelicerae and pedipalps) mass is smaller than the body mass (26% of total mass). Furthermore, flexibility of the body, particularly at the pedicel, provides additional movement of the centre of mass. CT data demonstrates that the complete removal of appendages from the model moves the centre of mass location in the sagittal plane 6% of the total body length posteriorly. On this basis, we chose to use an assumed fixed centre of mass location on the body based on nominal locations of the appendages and un-deflected body, see Fig. 1g and h. Previously published work on salticid jumps in another species has assumed the position of the centre of mass as the centre of triangle connecting the pedicel to both posterior lateral eyes^20^; our prediction of centre of mass is further aft of this position.

### Jumping experiment

**Figure 2:**
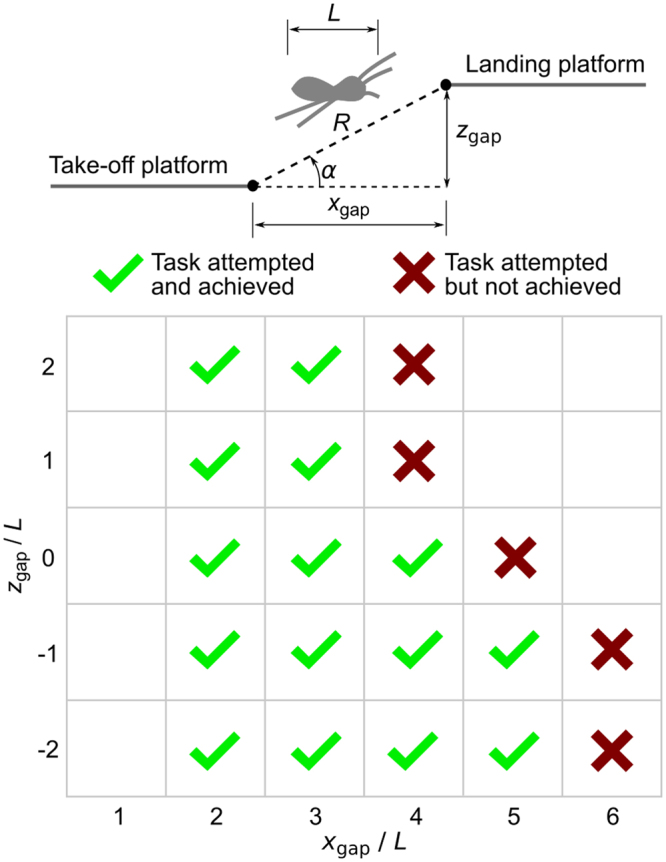
Definition of the jumping task and task test matrix. The jumping task is defined through the gap horizontal and vertical distances as ratios of the reference body length. Ticks indicate successful jumping attempts, whereas crosses demonstrate jumping tasks that the spider was presented with but refused to perform.

An experiment was designed in which motion of a spider could be recorded for a set of jumping tasks defined by horizontal platforms displaced horizontally and vertically in a two-dimensional plane, Fig. 2. Physical details of the experimental setup are given in the Materials and Methods section. The horizontal displacement was varied between 2 *L* and 5 *L*, and the vertical displacement between negative 2 *L* and positive 2 *L*, where *L* is the reference length (body length) of the spider. For convenience we will use a coordinate system (*x*_gap_, *z*_gap_) to refer to the location of the landing platform edge relative to the take-off platform edge. Jumps corresponding to positive, zero and negative vertical displacement are referred to as ascending, level and descending, respectively. Take-off is defined as the moment when all legs have left the take-off platform and the spider becomes fully airborne. For our purposes, we define the take-off point to be the location of the nominal centre of mass of the spider at the moment of take-off. We note that the natural behaviour of the spider is to start the jump close to the end of the platform. Thus at take-off the centre of mass of the spider is typically *forward* of the end of the take-off platform. Landing is defined as the point where the legs make first contact with the landing platform. Thus, the centre of mass of the spider can typically be *in front of* the proximal edge of the landing platform where the spider executes a minimum length jump. Whilst the true jump distance is thus typically not equal to the task distance defined by (*x*_gap_, *z*_gap_), we choose to use the task distance as the reference condition for ease of comparison between different jumps. For convenience, in what follows, we will refer to a jumping task for specific values of using *x*_gap_ and *z*_gap_ as ‘Task (*x*_gap_, *z*_gap_)’.

**Figure 3:**
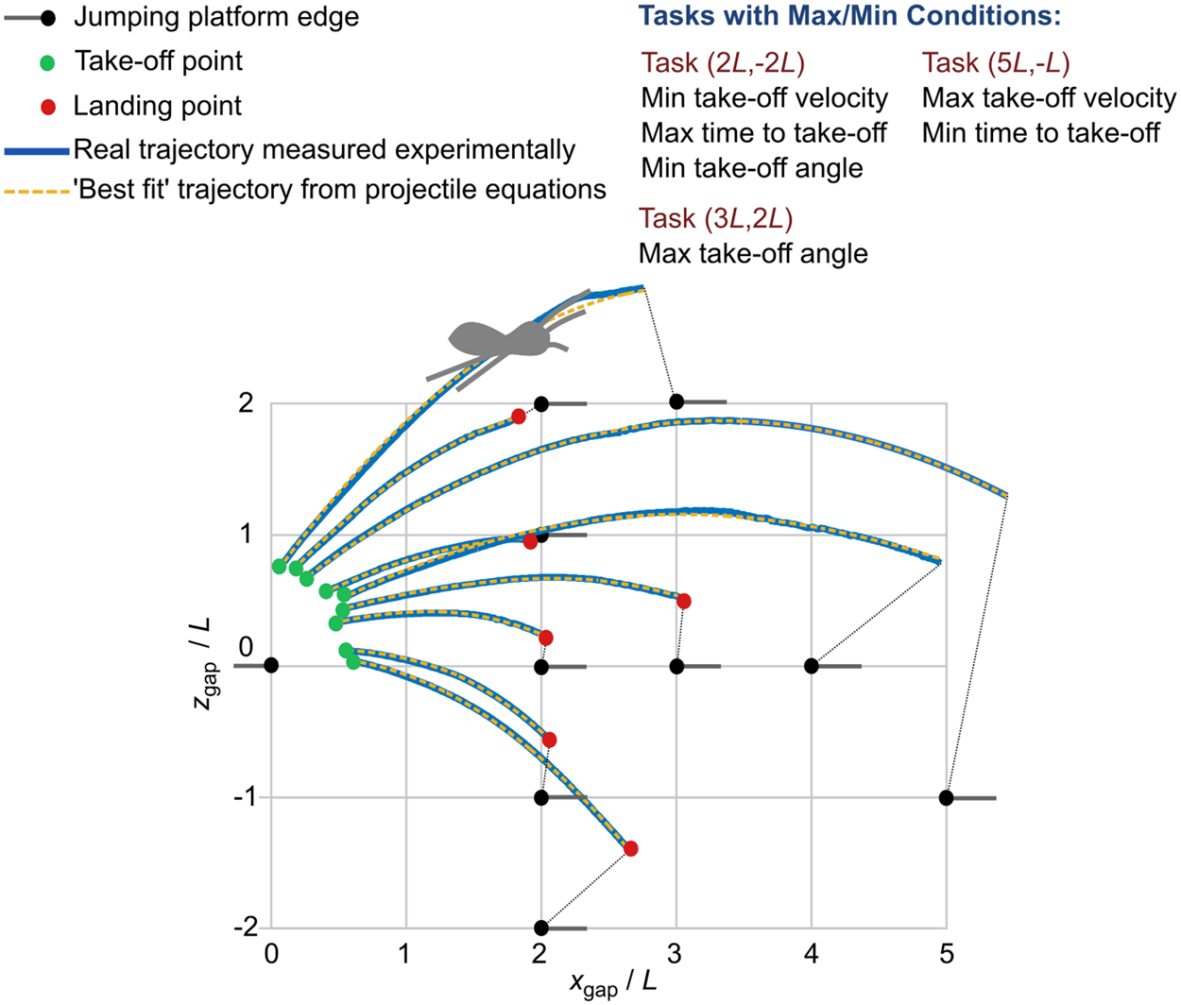
Example jump trajectories. Green circles indicate the location of the centre of mass at take-off. Red circles represent the location of the centre of mass at landing. No marker at the end of the trajectory indicates that the spider left the measurement field of view before landing. Leader lines from the ends of each trajectory indicate the landing platform location for that particular task. Background grid squares have dimensions *L* × *L* where *L* is the spider reference length (*L* = 15 mm). Blue lines are examples of the experimentally measured trajectories. Orange dashed lines are the calculated trajectories based on ‘best fit’ between projectile equations and experimental data.

**Figure 4:**
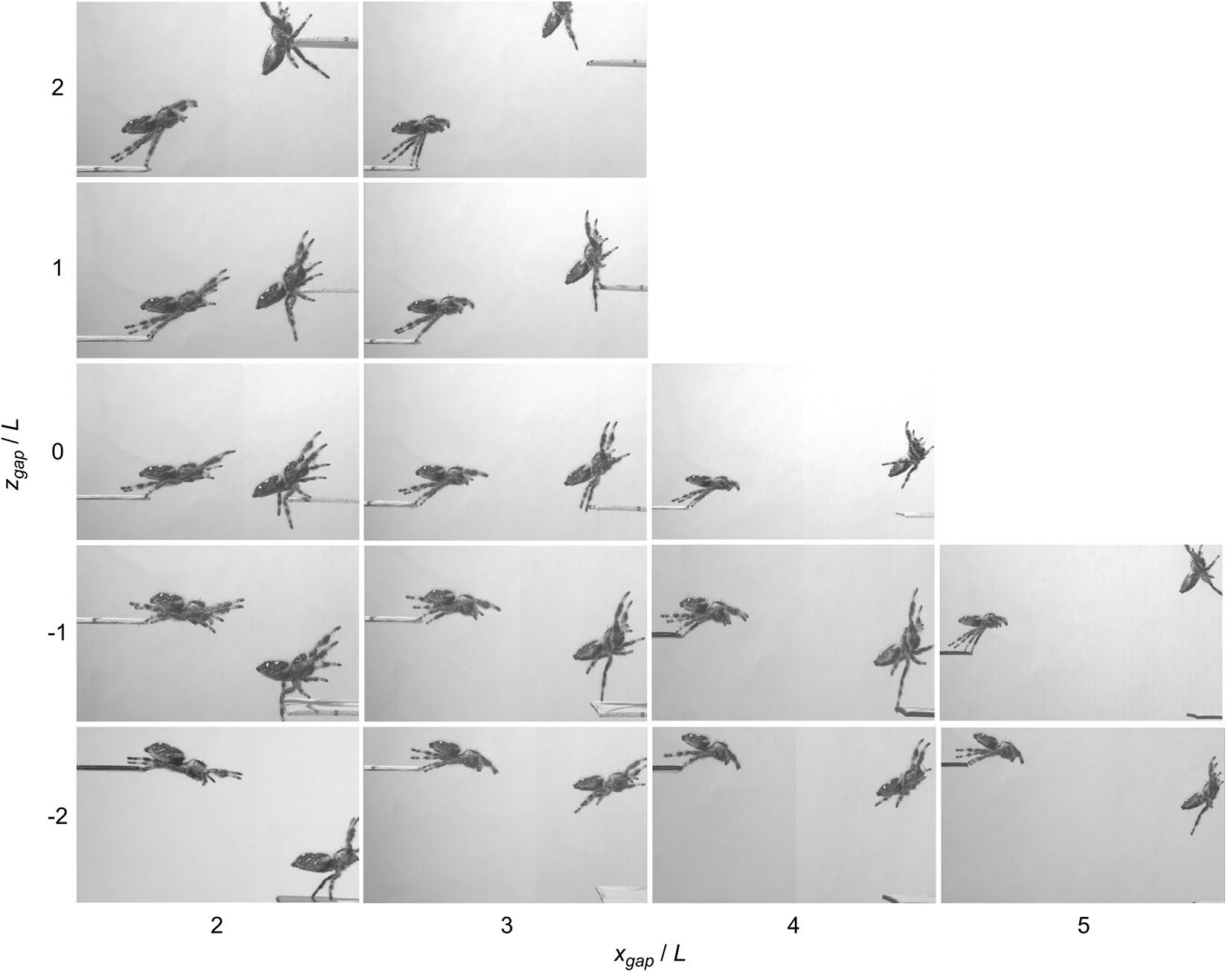
Visual comparison of body attitude and leg arrangement at the start and end of the jumping tasks. The starting frame is the instant of take-off. The end frame is taken either at the point where the spider makes contact with the landing platform, or where the centre of gravity passes the longitudinal location of the task landing point, depending on which happens first.

Four *P. regius* specimens were obtained for the experiment. However, it proved difficult to induce the spiders to jump in the test chamber and ultimately only one individual (female, body mass 150 mg, *L* = 15 mm) showed any inclination to jump as required, hence all experimental data is based on this individual. Time constraints on the experimental work meant that it was only possible to obtain a single example jump for a given jumping task (15 tasks/jumps in total). This reflects the difficulty in eliciting these sorts of activities in an experimental environment. The *P. regius* individual tested was capable of: (1) descending jumps up to 5 body lengths; (2) level jumps up to 4 body lengths; and (3) ascending jumps up to 3 body lengths. Figure 3 shows examples of the jumping trajectories where it can be seen that to minimise the jumping distance, the spider always starts at the edge of the platform and stretches its third and fourth legs so that its body is beyond the end of the take-off platform at the instant of take-off. Additionally, it can be seen from Fig. 3 that the spider changes its take-off point depending on the landing platform location. As expected, there is considerable variation in the landing position compared to the reference landing coordinates for different jumping tasks. For short jumping tasks, the actual landing point was close to the landing reference coordinates, but for long jumping tasks there were larger variations. To better demonstrate the take-off and landing behaviours, take-off and landing snapshots are shown in Fig. 4. Videos of the 15 jumping tasks are provided in Supplementary Movie S2.

Figure 4 and accompanying movie (Supplementary Movie S2) also allow several additional observations. First, for take-off: (a) the body attitude is dependent on the jumping task and (b) both third and fourth legs are used during jumping; however, their relative contribution to the force production could only be confirmed through experimental force plate measurement which is beyond the scope of the current work. Note that Parry and Brown^19^ suggest for *S. pubescens* that the third leg contribution can be neglected. Our videos show that the third leg is the last to leave the take-off platform. Second, depending on the task, the spider will have a different body posture at landing/approach: (a) for ascending jumps (*z*_gap_ > 0), it is clear that landings approach a vertical (upright) posture. (b) For level jumps (*z*_gap_ = 0), the ability to land at the target destination is evident for short jumps; however, the spider starts to overshoot by considerably longer amounts as the task distance increases. (c) For one body length descending and up to four body lengths horizontal jumping tasks (*x*_gap_ < 4 *L*, −*L*), landing is typically at or near the edge of the landing platform. (d) For two body lengths descending and up to four body lengths horizontal jumping tasks (*x*_gap_ < 4 *L*, −2 *L*), jumps tend to always overshoot the edge of the landing platform. This may be due to reduced precision of control for these more challenging jumps, or due to limited visual capabilities affecting the ability to correctly estimate the required trajectory. In either case, it appears that in the presence of uncertainty, the jumps tend to overshoot, which from a behavioural point of view is probably a better strategy than undershooting. (e) Finally, for descending jumps at five body lengths horizontal distance (5 *L*, *z*_gap_ < 0), the spider has very low precision, and its jumps appear rather haphazard. However, importantly, the spider does *attempt* this task. For a horizontal gap greater than 5 *L* the task is not attempted. Clearly the spider can jump this distance, but chooses not to, possibly for the simple reason that it cannot properly see the landing platform.

**Figure 5:**
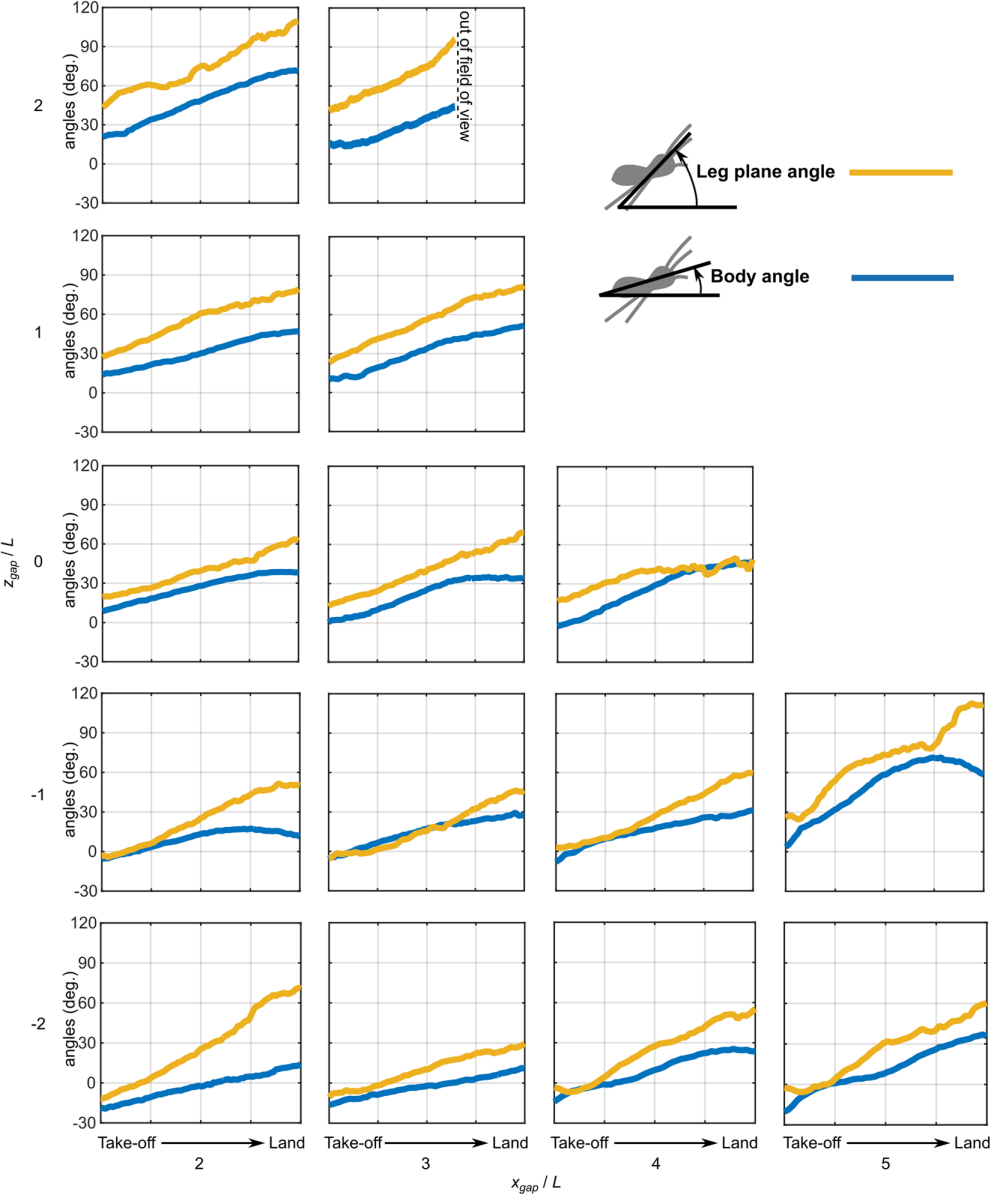
Variations of the body angle and leg plane angle from take-off to landing. Take-off and landing are as defined in Fig. 4.

In addition to these initial observations, our experiment allows us to conduct a quantitative analysis. Given the nature of the jumping motion, of relevance is both the pitch angle of the body *and* the pitch angle of the nominal plane of best fit through the tips of the spiders legs, which we shall call the ‘leg plane’, Fig. 5. The projection of the leg plane on the velocity vector of the spider is important because it defines the swept volume of the trajectory within which prey could be considered captured and/or substrate could be grasped to effect a landing. The definition of the leg plane is *necessarily* approximate because the tips of the legs do not ordinarily lie in the same plane; however, even an approximate estimation of this plane is of interest and since the angle of plane may vary by significant amounts during a jump, the effect of uncertainty in measurement is mitigated to some extent.

From the results presented in Fig. 5 it can be seen that the body angle at take-off varies depending on the jumping task. It is generally more positive (head up) for ascending jumps; this may be related to the limited field of view of the eyes and the need to face the jump target. The body attitude at landing is generally increased over the attitude at take-off, i.e. all jumps involve head up rotation (positive pitch) during flight. The leg plane angle is generally more positive than the body angle throughout the jump and generally increases at a greater rate, i.e. the relative angle between the body plane and the leg plane increases. The rate of change of the body and leg plane angles are approximately constant during the jump trajectory, hence the net torque acting on the overall spider is approximately zero. It should be noted that several insects are capable of aerial righting in mid-air, e.g. wingless pea aphids^27^ and wingless stick insect nymphs^28^. However, the time scale of the reported behaviours is significantly longer than that of the jumps reported herein and hence it is probable that aerial righting does not contribute significantly in the present case.

**Figure 6:**
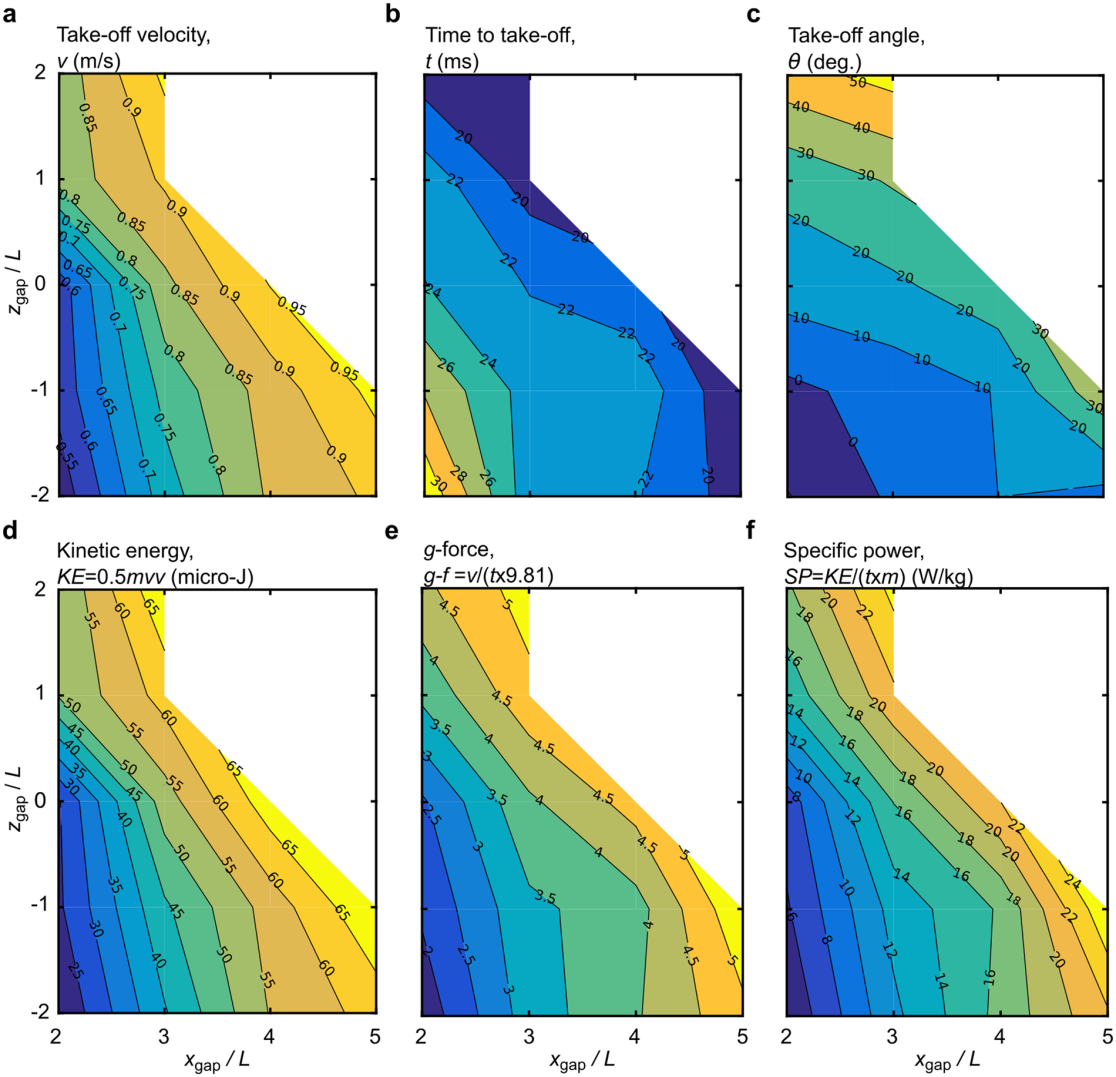
Contour plots of jumping characteristics of the *Phidippus regius* spider. (**a**–**c**) Main data extracted from the experiments: (**a**) The velocity magnitude at take-off, (**b**) time to take-off, and (**c**) trajectory angle at take-off. (**d**–**f**) Main jumping metrics used to assess the jumping performance: (**d**) Kinetic energy of the jump, (**e**) ratio of the jumping acceleration at take-off to gravitational acceleration (*g*-force), and (**f**) specific power of the jump relative to the total body mass.

We use the jumping trajectories for the 15 jumping tasks to analyse the jumping performance. Data for each of the 15 jumps are provided in Supplementary Dataset S3 and are plotted in Fig. 6. Dot-line plots of Fig. 6 are also provided as Supplementary Fig. S4. The principal measurements are of the take-off velocity, time to take-off, and the take-off angle. We then derive the kinetic energy, acceleration and specific power from the take-off velocity and the time to take-off. The velocity at take-off varies between 0.52 m/s and 0.97 m/s. Minimum take-off velocity was for task (2 *L*, −2*L*), and maximum take-off velocity was for task (5 *L*, −*L*). The time to take-off varies between 18.1 ms and 31.6 ms. Minimum time to take-off was for task (5 *L*, −*L*). Maximum time to take-off was for task (2 *L*, −2 *L*). Note that the jumping tasks with the maximum and minimum values of take-off velocity coincide with the minimum and maximum time to take-off, respectively. Analysis of the video data shows that the ‘stroke length’ of the jump (the linear distance over which the extending legs do inertial work on the body) is approximately independent of take-off speed, see Supplementary Fig. S5. Assuming a simple constant acceleration model, the take-off speed, *v*, time to take-off, *t*, and the stroke length, *s*, are related as *v* = 2 *s*/*t*. If the stroke length remains constant, then *v* and *t* are inversely related consistent with the experimental observation made above. The take-off angle varies between −10.5 degrees and 53.5 degrees. Minimum take-off angle was for task (2 *L*, −2 *L*). Maximum take-off angle was for task (3 *L*, 2 *L*). The trajectories for minimum/maximum take-off velocity, time to take off and take-off angles are shown in Fig. 3. Generally, as shown in Fig. 6a,c the velocity at take-off and the take-off angle will increase as the horizontal gap increases and the vertical gap becomes more positive. The kinetic energy is derived from the velocity at take-off hence correlation is implicit. The minimum and maximum values of acceleration are 1.68 g and 5.43 g, and the variation is similar to that for specific power. In general, the variations of kinetic energy, acceleration, and specific power all will increase as the horizontal gap increases and the vertical gap becomes more positive.

Measured jumping trajectories matched closely with ballistic trajectories predicted from initial conditions at take-off; see examples provided in Fig. 3. The forces acting on the spider during flight are due to gravity, air resistance and silk line tension^22^. The silk line force is considered negligible^29^. As a conservative estimation of the drag force, we assume that the spider is sphere of diameter equal to the body reference length. The drag coefficient calculated based on the relation recommended in^22,30^ is approximately 0.63 ± 0.03 (see Supplementary Dataset S3). Based on these values, the maximum aerodynamic drag for the highest speed take-offs is less than 4.2% of the weight of the spider (see Supplementary Dataset S3), and hence we also assume to be negligible.

To better understand the jumping behaviour of *P. regius*, it is instructive to compare the jumping results against some reference optimal conditions. Here, maximum travel distance (related to minimum energetic cost of transport) and minimum travel time for a given power budget are considered. Expressions to evaluate the trajectory take-off angle corresponding to both optimum conditions are derived based on^5^ and final forms of the governing equations are as follows:

For maximum travel distance:

1 $$2{\theta }_{\text{max}{\rm{d}}{\rm{i}}{\rm{s}}{\rm{t}}{\rm{a}}{\rm{n}}{\rm{c}}{\rm{e}}}=90+\alpha $$

**Figure 7:**
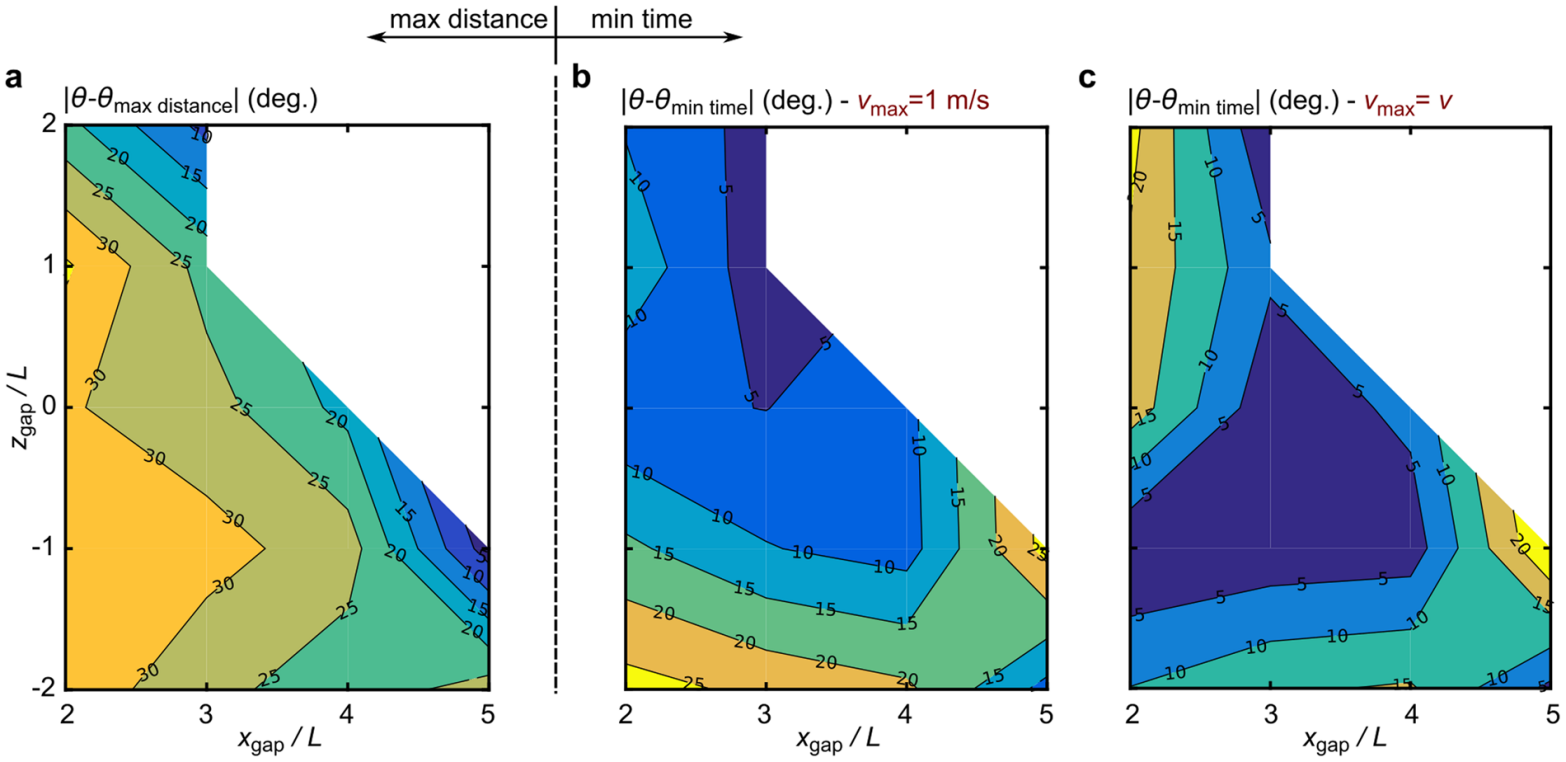
Contour plots for comparison of measured take-off angles for different jumping tasks with the theoretical take-off angles required for (**a**) minimum cost of transport and (**b**,**c**) minimum time of flight. Plots show the magnitude of the difference in angle for clarity of interpretation. In each case darker colours correspond to jumping at closer to the respective optimum conditions. In plot (**b**), minimum flight time angles are computed based on a reference take-off velocity of 1 m/s. In plot (**c**), minimum flight time angles are computed based on a reference take-off velocity equal to the take-off velocity of each task.

For minimum travel time:

2 $$\cos \,{\theta }_{\text{min time}}\,\sin (\alpha -{\theta }_{\text{min time}})=\frac{-9.81R{(\cos \alpha )}^{2}}{2{v}_{max}^{2}}$$

where $\theta$ is the take-off angle and $\alpha$ and $R$ are defined in Fig. 2. Note that equations (1) and (2) describe the optimum take-off angles for the assumption of a ballistic trajectory. For the maximum distance, the optimum angle identified *is by definition* the least energy consuming jump among the many ways to reach that goal. Figure 7a shows the deviation of the measured jumping angle at take-off from the optimum take-off angle for maximum travel distance calculated using the model. This comparison supports our hypothesis, demonstrating that as the jumping challenge becomes harder (typically ascending and further jumps), jumps approach energy optimum conditions; i.e. the spider is forced to jump efficiently.

The expression for minimum time represented by equation (2) requires a reference velocity, *v*_max_. Two approaches can be used to select this reference velocity. Our first approach is to use a single *absolute* maximum velocity corresponding to the maximum energetic capability of the spider. This provides an objective comparison of all tasks against what would be possible if the spider always jumped at its maximum speed. However, this comparison becomes less meaningful when jumps are not made at the maximum speed. The second approach is to use the maximum jump velocity for each task as the reference velocity (which in our case means that *v*_max_ = *v*). This provides a more representative test of time optimality for jumps where the take-off velocity is much less than the absolute maximum velocity, but is arguably weaker overall in that it does not provide a comparison with the global minimum time possible.

Figure 7b uses the first approach to show the deviation of the measured jumping angle at take-off from the optimum take-off angle for minimum travel time. The reference maximum take-off velocity used here is equal to 1 m/s based on the take-off velocity values shown in Fig. 6a. The second approach is shown in Fig. 7c. Comparison of Fig. 7b and c shows that both approaches give a similar outcome: *P. regius* tends to adopt a minimum travel time jump for shorter jumping tasks. This again supports our hypothesis that for short distances, time of flight is prioritised. Note that dot-line plots of Fig. 7 are also provided as Supplementary Fig. S6.

There might be a concern on the value used within Fig. 7b to represent the maximum take-off velocity of this species. However, the suggested 1 m/s is the maximum value observed in our experiments and is very close to the mean value for maximum take-off velocity for the “muscle contraction” group explained in the next section. More importantly, the conclusion from Fig. 7b is only weakly sensitive to the exact value of *v*_max_ within its expected values. This fact is illustrated through comparing Fig. 7b for different *v*_max_ values, see Supplementary Fig. S7.

### Jumping performance comparison

We believe it is instructive to compare the jumping performance of *P. regius* against the performance of other spider species reported in the literature, and other jumping arthropods in general. Table 2 compares *P. regius* against two other spiders (*S. pubescens*^19^ and *P. princeps*^20^) for which full data of their jumping performance exists. Note that in Table 2, we are comparing the performance of *level* jumps that achieved maximum travel distance. *P. princeps* is similar to *P. regius* in terms of morphology, body mass and scale. In contrast, *S. pubescens* is an order of magnitude smaller than the other two species. Despite these differences in mass and scale, all three species are very close in terms of *g*-force at take-off and specific power which are the performance metrics most relevant to jumping. It is also worth noting that *P. princeps* and *P. regius* have similar velocity and trajectory angle at take-off, whereas it is reported that *S. pubescens* jumps a similar distance but with a lower take-off velocity and a flatter trajectory which is physically impossible (the ballistic equations would predict a jump distance of 18.6 mm for this combination of take-off angle and take-off velocity). However, there is some uncertainty of the exact travel distance reported for *S. pubescens* in^19^ which may explain this discrepancy.

**Table 2:** Comparison of the jumping performance of *Phidippus regius* against *Sitticus pubescens*^19^, and *Phidippus princeps*^20^. All three jumps are level jumps.

*Species*	Reported jump gap, *R* (mm)	Body mass, *m* (mg)	Take-off velocity, *v* (m/sec)	Time to take-off, *t* (ms)	Take-off angle, *θ* (deg.)	Acceleration, *a* (m/sec^2^) [*a* = *v*/*t*]	*g*-force, *g-f* [*g-f* = *a*/9.81]	Kinetic Energy, *KE* (μJ) [*KE* = 0.5*mv*^2^]	Jumping Power, *P* (mW) [*P*=*KE/t*]	Specific Power, *SP* (W/kg), [*P*/*m*]
*Sitticus pubescens*	~50	10	0.67	13.06	12	51.30	5.23	2.24	0.17	17.19
*Phidippus princeps*	60	150	0.83	16.15	22	51.41	5.24	51.67	3.2	21.33
*Phidippus regius*	60	150	0.95	20.63	26.50	46.16	4.71	67.97	3.3	21.97

The jumping performance of spiders is comparable to that of jumping insects. Following the classification proposed by Burrows and Dorosenko^31^, we classify jumping insects into two main categories: (1) those expected to employ *muscle contraction* for jumping, and (2) those thought to employ a *catapult mechanism*. This is based on the assessment of the muscle specific power for a species and comparing it to the contractile limits of muscle, which ranges between 250 up to 500 W/kg^31–35^. Here, we assume that all insects have the same propulsive leg muscle mass to body mass ratio of 10%^31,36–40^. If the majority of species for one insect group lies below the 500 W/kg limit, then they are classified as relying on muscle contraction, otherwise they are considered to use a catapult mechanism. Clearly, this classification does not stop the possibility of an insect employing either mechanism, or a combination of both. Nonetheless, this exercise remains instructive because it provides a logical criterion to classify jumping.

**Figure 8:**
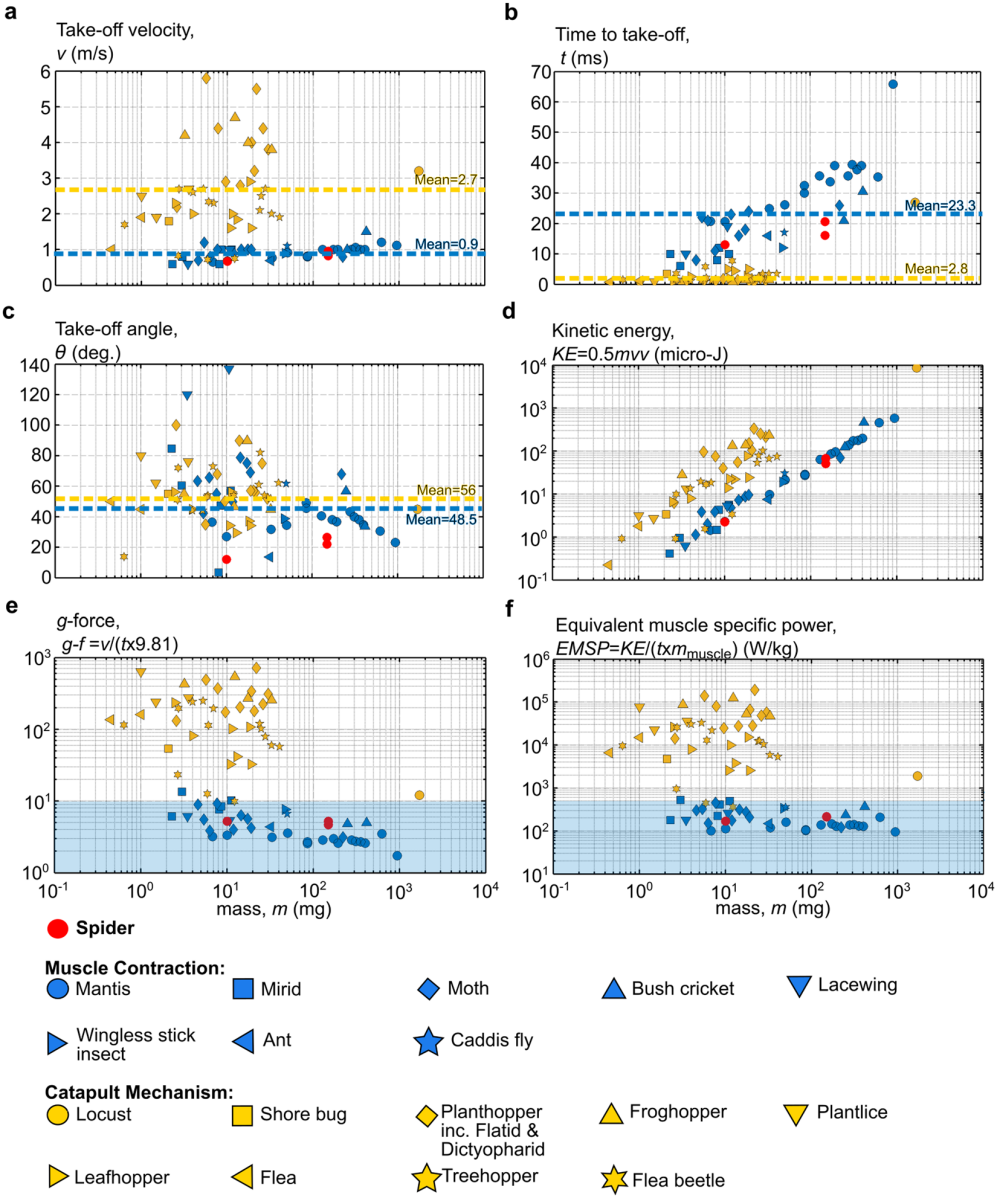
Comparison of the jumping characteristics of the different insects. Spiders are highlighted with red circles. Insects are classified into two main groups: a muscle contraction group and a catapult mechanism group. The sub-figures are: (**a**) The velocity magnitude at take-off, (**b**) time to take-off, (**c**) trajectory angle at take-off, (**d**) kinetic energy of the jump, (**e**) the ratio of the jumping acceleration at take-off to gravitational acceleration (g-force), and (**f**) the specific power of the jump relative to the muscle mass. Full data and supporting references are provided in Supplementary Dataset S3.

Figure 8 provides the jumping characteristics for the different insect groups constructed for this study. The data upon which Fig. 8 has been produced are provided in Supplementary Dataset S3. Figure 8 allows us to compare spider jumping performance metrics against those of other species employing different actuation strategies and hence allows us to comment on the ongoing debate of the role of hydraulic versus muscular actuation for the present spider species. Values shown in the figure correspond to the jump with highest take-off velocity recorded in the literature (sometimes this is an individual jump, but on other occasions it is a mean value if that is all that is available). Eight insect groups (36 different species) fall within the muscle contraction limits, and nine insect groups (42 different species) fall within the catapult category. From this exercise, we draw several observations: first, insects from the muscle contraction group have maximum take-off velocities clustered around the 1 m/s value we saw in our spider; in contrast, insects employing a catapult mechanism have greater variability in take-off velocity. Whilst the maximum take-off velocity for this latter group could reach 6 m/s (in plant-hoppers), the mean for the group as a whole is three times that of the muscle contraction group. Second, the mean value of the time to take-off for the catapult group is around an order of magnitude less than the muscle contraction group. Third, there is a similar wide variation of take-off angle for both groups, with average of around 45 degrees. Finally, *g*-force is strongly correlated with specific power, and hence *g*-force may be used to differentiate between the two groups. As shown in Fig. 8e, an insect is within the muscle contraction group if the *g*-force is below 10.

Spiders clearly fall within the muscle contraction group: values for take-off velocity and time to take-off are very close to the group mean. On the other hand, the take-off angle for spiders is notably lower than for the insects in both groups; salticids prefer shorter, quicker paths rather than energetically optimal paths, presumably related to their predatory life style (see Fig. 7). This does not necessarily imply that spiders use muscle contraction whilst jumping. However, it is evident from the above analysis that muscles alone could actuate their jumps.

## Discussion

Our results show that *P. regius* uses a range of different jumping strategies depending on the jumping task. It uses flat trajectory jumps for short distances whereas it uses steeper jumps close to the energetically optimum trajectory at longer distances. This is in line with findings from other jumping studies^5,41^ where the choice of jumping trajectory is altered depending on both the mechanical limitations and the ecological function of the jump. For long distance jumps the power limitations of the spider may force it to use the distance optimum trajectory, whereas for shorter jumps it can choose from a range of available take-off angles, and because these short jumps are typically used for prey capture, a rapid low trajectory may well be the best choice. There may also be sensory reasons for the choice of jump trajectory. Jumping spiders have good eyesight for arachnids, with four large anterior facing eyes, but these eyes are not mobile, have narrow, non-overlapping fields of view and it has been suggested that the spider uses a system of image defocus to judge distance^42^. This may well constrain the starting posture of the jump since the animal needs to be able to see the target, and may discourage steep trajectories where the target would not be in sight throughout the jump.

It is clear from our results that whilst *P. regius* is a competent jumper, its maximum jumping distance is limited compared to other arthropods. This may be expected, because this species uses precise jumps for prey capture, and not primarily as an escape response which seems to be the main purpose for many of these extreme jumps. In addition, the animal is apparently not able to perform the full range of jumps that would be predicted mechanically (i.e. there are some combinations of height change and distance that are possible given its leg power, but that are not used). This could be a sensory limitation rather than a mechanical one, as mentioned above. Equally, this may be a behavioural constraint - the animal may simply have been unwilling to perform these particular combinations of distance and height change. It is always impossible in animal locomotion experiments to define the absolute limits of what the animal is physiologically able to perform and certainly in our case it is very likely that we were not seeing the very longest jumps. In addition, it must be remembered that our results reflect the jumping abilities of a single spider. It is entirely possible that these results are not representative of the species as a whole which means that caution must be taken when interpreting our data. However our results certainly document in detail the jumping abilities of this individual and it is likely that jumping in spiders is a relatively stereotyped activity such that we would not expect a great deal of difference between individuals in their jumping kinematics. In particular, mechanical constraints that affect jumping capabilities directly should be apparent in all individuals.

Our results also allow us to calculate the power requirement for the jumps observed and based on our comparisons with the jumps performed by other arthropod groups, there would not appear to be any need for an elastic storage mechanism since the power requirements could be achieved using available muscles. Equally there is no need to suggest that propulsive power for jumping is generated using haemolymph pressure and it is possible that in this species the primary propulsion is generated by the leg muscles directly (although probably it is more likely to be a combined system as previously suggested^17^). Without dynamic pressure measurements and/or direct measurements of muscle force generation it is not possible to identify the source of the motive force; however, this needs to be pursued in future work. *P. regius* apparently uses the back two pairs of legs for take-off and this may reflect a greater reliance on leg muscle for powering take-off compared to species that use only a single pair of legs^19^.

The mid-air and landing postures used by the spider are also of note: it clearly deploys its legs differently depending on the nature of the target. Furthermore, the spider starts to considerably overshoot as the jumping distance becomes larger. This may be because of poorer visual discrimination at longer distances or the inability to execute accurate motion control at very high accelerations. Jumping animals employ a range of different strategies to control their body orientation in mid-air. These include mechanisms based on altering aerodynamic forces or exchanging angular momentum between rotating body parts^43^. Jumping spiders are notable for their silk line which may contribute to body orientation control in flight for some spider species (e.g.^22^). In our spider, there was a continuous adjustment of leg position during the flight, however we found no strong evidence that the silk thread contributed to the dynamics during the jump (the trajectories of the animal were always ballistic). This would suggest that in this species the silk thread is not used to orient the body during the aerial phase, and is therefore more likely to simply act as a safety line in case of a missed landing. We did not, over the course of this study, observe an aborted or failed jump, although there were occasions when the landing posture could be described as inelegant. Generally, landing mechanics are far less well studied than those used in take-off, and this is again an area that would benefit from further study.

Design and build of bio-inspired jumping robots continues to be an area of interest. Examples include a flea inspired jumping mechanism^44^, grasshopper^45^ and galago^46^ inspired jumping robots among several other examples. These designs typically employ an elastic element to modulate power and achieve the highest possible jumps. However, less attention has been directed towards creating spider-inspired jumping robots which prioritise speed and accuracy over jumping distance. Our study highlights that under some circumstances optimality with respect to time may be more important than distance. Furthermore, our results show that spiders can accurately plan jumps to achieve a specific landing target. This is in line with studies of insects that demonstrate accurate motion planning^43,47^. A number of studies report spider-inspired jumping robotics in the literature. Shield *et al*. developed a LEGO robot to study the effect of a silk line on pitch control^48^. Faraji *et al*. built a jumping mechanism to research the effect of spider front leg orientation on jumping trajectory^49^. Most recently, Sprowitz *et al*. demonstrated a spider-inspired pneumatic leg joint^50^. Whilst these studies provide an improved understanding of engineered spider mechanisms, generally, there has been little attention to the overall problem of sensing, control and actuation needed to undertake precise jumps in uncertain environments.

All in all, our results suggest that the form of jumping in these spiders is rather different from those seen in the wider arthropod community, and reflects the specialisations for short-range high speed jumps. We thus suggest there are strong behavioural and sensory constraints on the jump trajectory, whilst leaving questions about control and actuation open.

## Materials and Methods

### Spiders

Four female *P. regius* spiders were sourced from Urban Jungle pet-shop in Manchester. Each spider was kept individually in captivity at constant room temperature of 22.5 °C. They were constantly supplied with water and fed with one cricket a week. One spider was inclined to jump within the experimental apparatus; the other three were unwilling to jump. All jumps were collected within a week. Body mass was monitored on daily basis and confirmed as approximately constant. We did not use prey as a bait item as this would mean one jump per week based on the observed diet of the spider. Instead, the spider was manually transported between the take-off and landing platforms until it became familiar with the challenge. No form of stimulation (e.g. air blowing) was used to induce a jump. The spider did not fail any of the jumps; it was either a jump or no-jump situation. Hence, once a jump was performed it was deemed a successful trial.

The animals used in the present study were handled in accordance with institutional guidelines for the use of Animals in Research. UK law does not require special permits for the use of arthropods in research.

### CT-scan

A female *P. regius* was scanned using a Nikon HMX-ST 225 system at the Manchester X-ray Imaging Facility. This employed a molybdenum reflection target, a current/voltage of 260 µA/60 kV. No filter was added, and 5013 projections at 708 milliseconds exposure were collected. The 3192 × 2296 detector panel allowed a volume with 7.2 μm voxels to be created using CTPro V4.3. The volume was loaded in the open source software Drishti^26^, and non-linear scaling applied to the histogram prior to binning and export as an 8-bit PNG image stack. This stack was imported into the SPIERS software suite^25^, where a linear threshold was applied, the limbs were manually segmented, and the stack was visualised by using isosurfacing to generate a triangle mesh. The CT results are provided as Supplementary Files S1 in the VAXML interchange format^25^, as a Drishti Prayog model^26^ for public engagement and data exploration, and as the original volume file created by CTPro. Measurements were derived from the VAXML model within SPIERSview. The surfaces of this were imported into the open source package Blender following the methods of Garwood and Dunlop^51^, and publication quality images (Fig. 1) and a Movie (Supplementary Movie S2) were rendered using raytracing.

### Jumping platform

**Figure 9:**
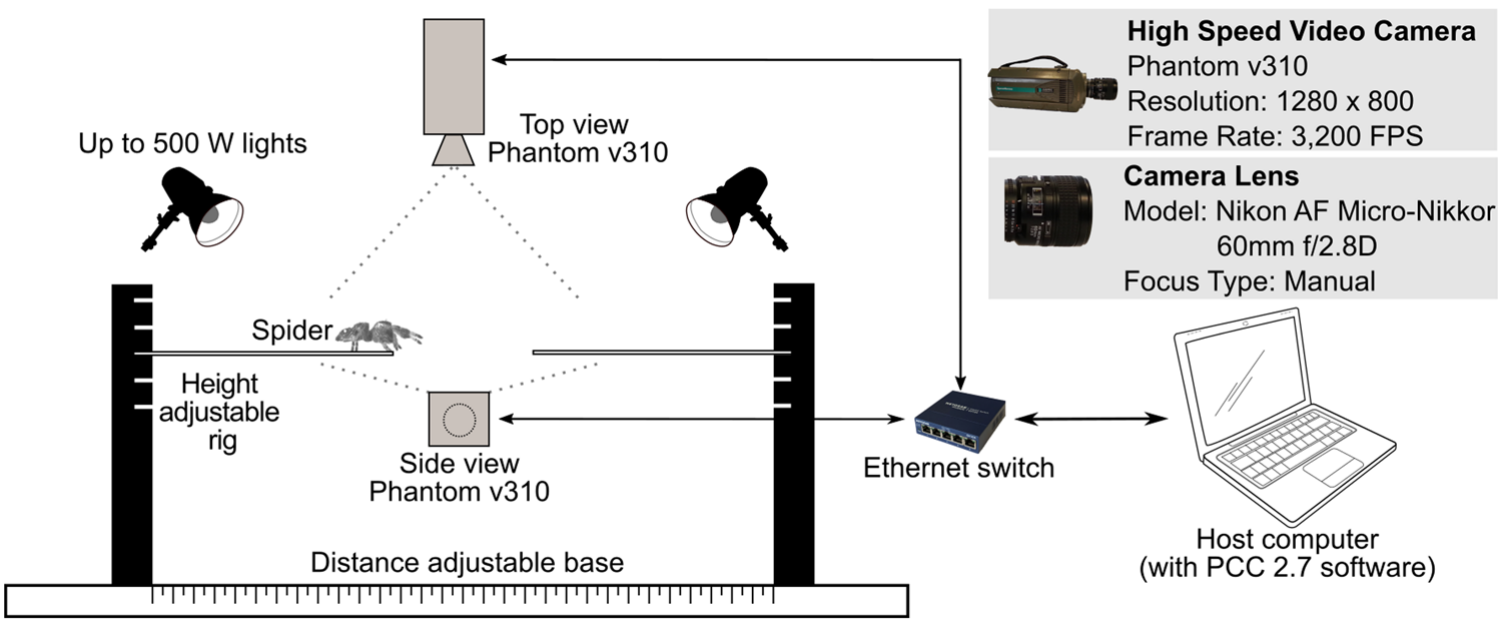
The jumping experiment set-up.

A jumping platform was designed to conduct the experiments as shown in Fig. 9. The take-off and landing platforms were approximately 200 mm above the ground. Platforms were made from plywood with dimensions 100 × 20 × 1 mm (length, width, thickness). Cut edges were sanded smooth but take-off and landing surfaces were otherwise left in the as supplied state to provide a slightly rough surface that the spider could grip easily. The take-off is consistently from a uniform horizontal ground reference, as opposed to more complex geometries involving surfaces with vertical components, e.g. from edges. This was achieved by making the platform sufficiently low thickness (<0.1 *L*) that the spider favoured using the horizontal surface of the platform to jump from rather than the vertical surface at the end of the platform which occurred in an earlier version of the apparatus with thicker platforms. The jump was recorded using two ultra-high speed cameras (Phantom v310) operating at 3,200 frames per second with 1280 × 800 resolution and an exposure time of 310 μs. Both cameras were fitted with Nikon AF Micro-Nikkor 60 mm f/2.8D lenses. The top camera was mainly for exploring the motion of the leg segments in the transverse plane (not considered in this study); however, all the relevant jumping data presented in this study are based on the videos from the side view. Two 500 W halogen lights were used to improve the quality and clarity of the high speed videos.

### Kinematics acquisition and trajectory reconstruction

The camera was controlled using the software Phantom Camera Control PCC 2.7. Once a video was captured, the degree of brightness/contrast is adjusted for improved clarity. A calibration process was then conducted using several reference dimensions within the frame. The nominal centre of mass was tracked using the custom built function within PCC 2.7. Data for the trajectory, i.e. variation of the *x* and *z* coordinates in time were then exported for processing in Matlab (www.mathworks.com). The processing of the trajectory data allowed definition of the velocities and angles along the trajectory from which the velocity and angle at take-off values were identified for each jump. Checks were conducted to make sure that the obtained velocity and angle values at take-off are robust. This was done by identifying the ballistic velocity and take-off angle that would allow minimum Root Mean Square Deviation between the real trajectory and the theoretical ballistic trajectory. Hence, ‘best fit’ velocity and angle values at take-off for all 15 jumping tasks were identified, and are provided in Supplementary Dataset S3.

### Data availability

All data generated or analysed during this study are included in this published article (and its supplementary information files).

## Electronic supplementary material

Supplementary Information Files

Supplementary Movie S2

Supplementary Dataset S3
