# Supplementary material for: Energy and time optimal trajectories in exploratory jumps of the spider Phidippus regius
Source: Sci Rep. 2018 May 8;8:7142. doi: 10.1038/s41598-018-25227-9 (PMC5940701; doi:10.1038/s41598-018-25227-9)
Supplement: Supplementary file 1 — Supplementary Information Files [file 41598_2018_25227_MOESM1_ESM.pdf]

Supplementary Information Files for the Paper:

Energy and time optimal trajectories in exploratory jumps of the spider *Phidippus regius*

Authors: Mostafa R. A. Nabawy[1], Girupakaran Sivalingam[1], Russell J. Garwood[2,3], William J. Crowther[1], and William I. Sellers[2]

[1] School of Mechanical, Aerospace and Civil Engineering, The University of Manchester, Manchester M13 9PL, UK

[2] School of Earth and Environmental Sciences, The University of Manchester, Manchester M13 9PL, UK

[3] Department of Earth Sciences, The Natural History Museum, Cromwell Road, London SW7 5BD, UK

Supplementary Files S1: CT scan files. A repository with files from CT scanning composed of: (1) The volume from the CT scan in VGI/VOL format. (2) A VAXML model of the spider (stl meshes tied together with an XML file). (3) A Drishti Prayog model of the volume. Supplementary Files S1 can be accessed on Zenodo with the DOI 10.5281/zenodo.842844. These files can also be accessed by using the following unique and anonymous link:

https://zenodo.org/record/842844?token=eyJhbGciOiJIUzI1NiIsImV4cCI6MTUxMzg5MzU5OSwiaWF0IjoxNTAzNDEyOTg4fQ.eyJkYXRhIjp7InJlY2lkIjo4NDI4NDR9LCJpZCI6MjI2LCJybmQiOiIwZTNkZmYwOCJ9.QZOl-NIPTlbRrfuvZHAtKw2g5rMYtyqZWkylh338cpU

Supplementary Movie S2: A collection of videos including the CT scan result and jumps of the spider. Attached movie contains the CT scan model rendered within Blender as well as videos of the 15 jumping tasks performed by *Phidippus regius.*

Supplementary Dataset S3: Full data of the jumping characteristics of spiders and other insects. Attached Excel file contains 4 data sheets for: (1) Jumping data for each of the 15 jumping tasks performed by *Phidippus regius*. (2) Comparison of the jumping performance of *Phidippus regius* against other spiders. (3) Jumping data for the 'muscle contraction' group. (4) Jumping data for the 'catapult mechanism' group.

Supplementary Figures S4, S5, S6 and S7: Four additional supportive figures; please see below.

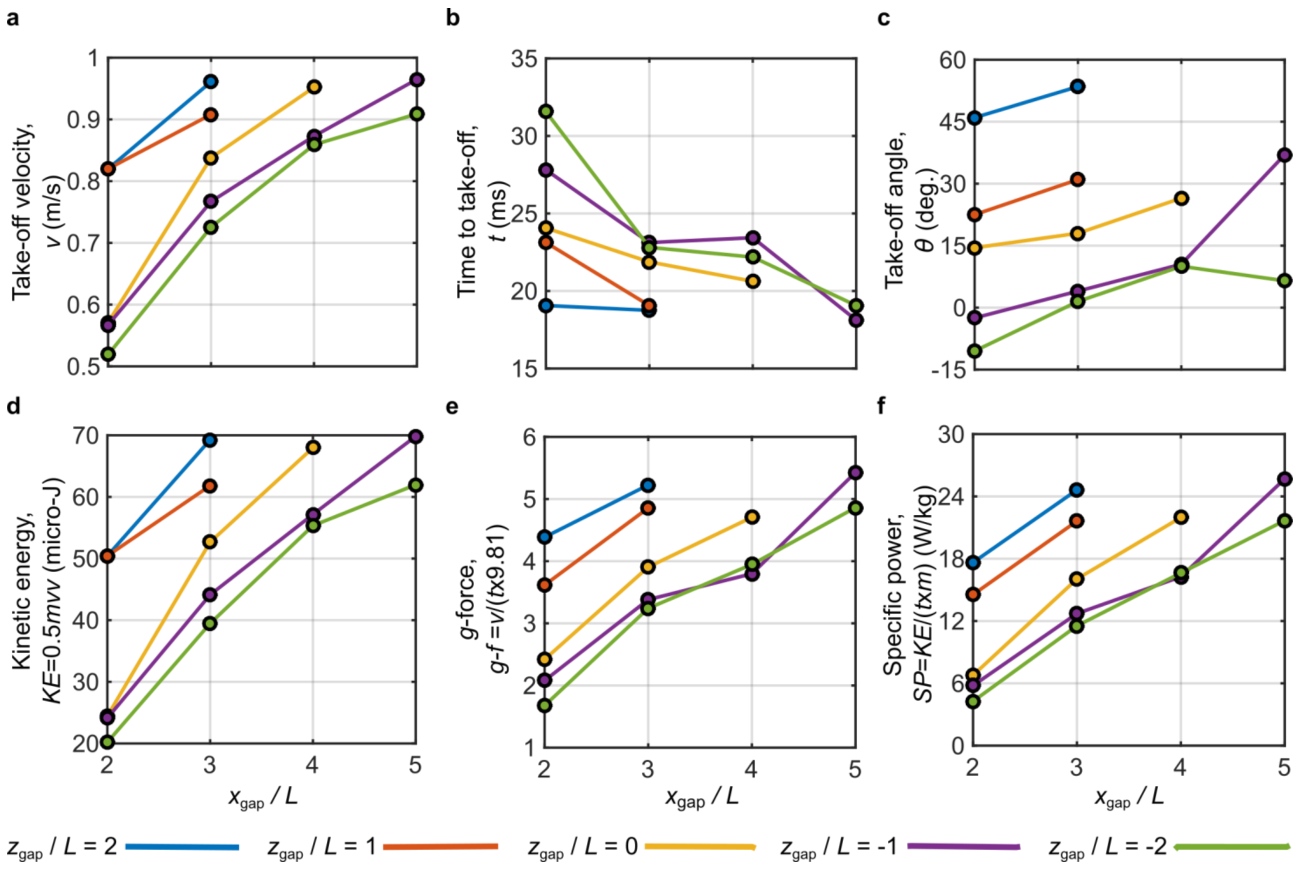

Supplementary Figure S4: Jumping characteristics of the* Phidippus regius *spider presented in alternative plotting format. Data the same as for Figure 6 of the main paper. **(a-c)** *Main data extracted from the experiments:* **(a)** *The velocity magnitude at take-off,* **(b)** *time to take-off, and* **(c)** *trajectory angle at take-off.* **(d-f)** *Main jumping metrics used to assess the jumping performance:* **(d)** *Kinetic energy of the jump,* **(e)** *ratio of the jumping acceleration at take-off to gravitational acceleration (g-force), and* **(f)** *specific power of the jump relative to the total body mass.*

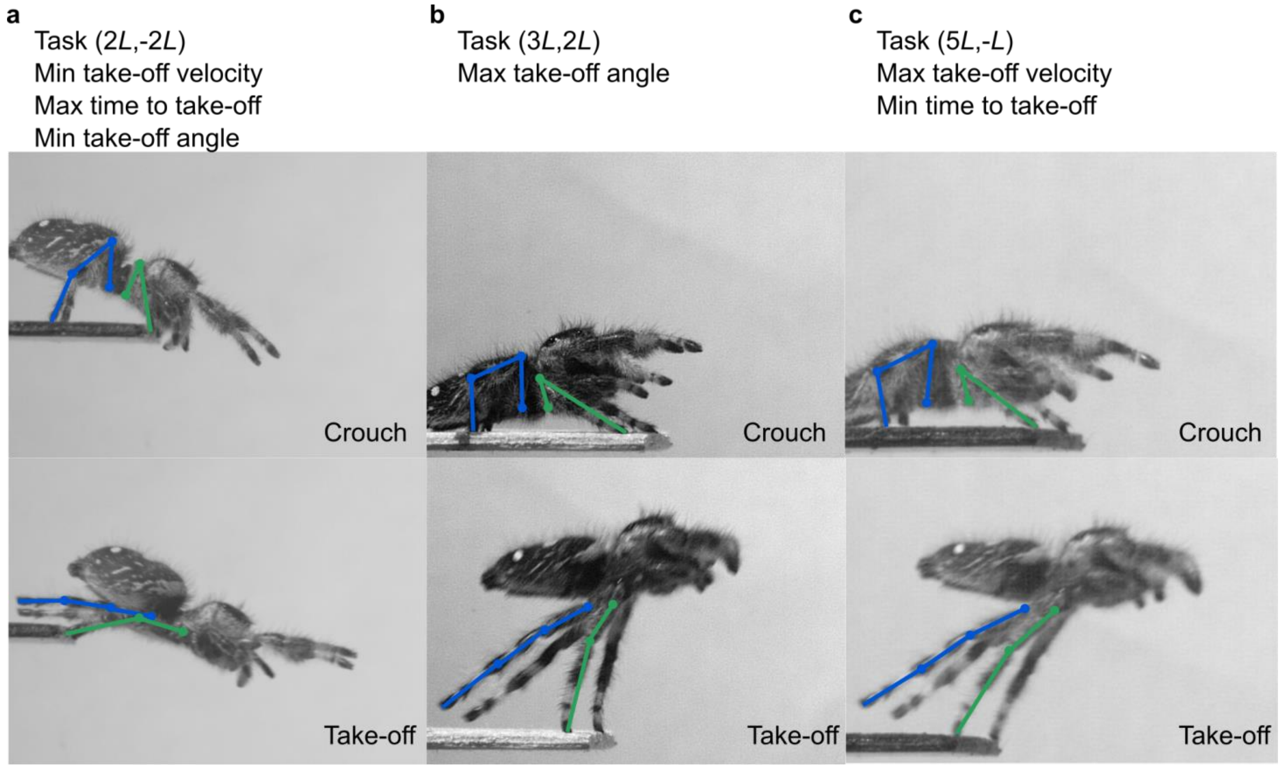

Supplementary Figure S5: Visual comparisons of leg straightening at crouch and take-off for maximum condition tasks. *It can be seen that spider applies the same form of leg motion within its jumps.*

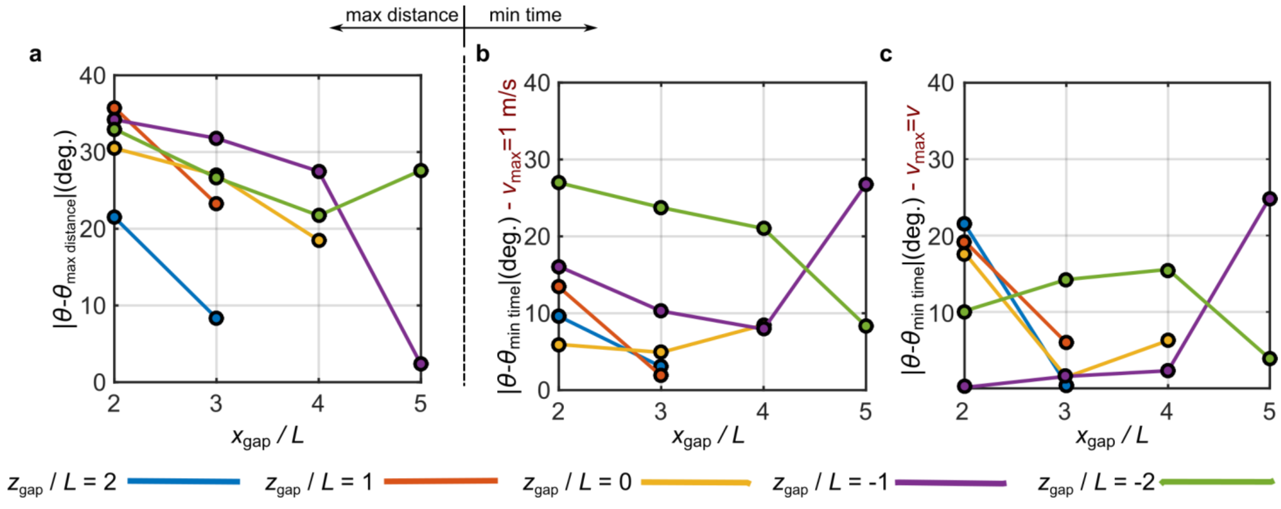

Supplementary Figure S6: Comparison of measured take-off angles for different jumping tasks with the theoretical take-off angles required for* (a) *minimum cost of transport and* (b, c) *minimum time of flight. Alternative plotting format. Data the same as for Figure 7 of the main paper. *Plots show the magnitude of the difference in angle for clarity of interpretation. In plot (b), minimum flight time angles are computed based on a reference take-off velocity of 1 m/s. In plot (c), minimum flight time angles are computed based on a reference take-off velocity equal to the take-off velocity of each task.*

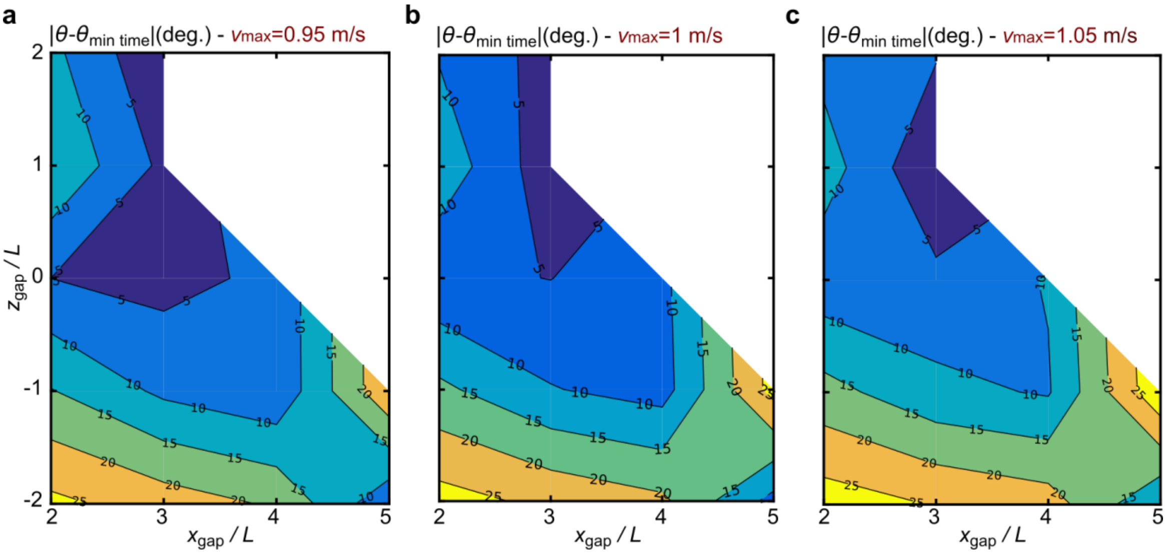

Supplementary Figure S7: Effect of the maximum take-off velocity value on minimum time of flight. **(a)**, **(b)** *and* **(c)** *represents maximum take-off velocity conditions of 0.95 m/s, 1 m/s and 1.05 m/s, respectively. Plots show the magnitude of the difference in angle for clarity of interpretation. In each case darker colours correspond to jumping at closer to the respective optimum conditions. The value of maximum take-off velocity is varied within its expected values showing that the conclusion from this demonstration is not strongly sensitive to this value.*
